# Extremity and Mandibular Reconstruction After Gunshot Trauma—Orthoplastic Strategies from Five Years of Humanitarian Missions in a Resource-Limited Setting

**DOI:** 10.3390/jcm14144852

**Published:** 2025-07-08

**Authors:** Viktoria Koenig, Tomas Kempny, Jakub Holoubek, Tomas Votruba, Julian Joestl

**Affiliations:** 1 University Clinic of Plastic, Aesthetic and Reconstructive Surgery, Medical University of Vienna, Währinger Gürtel 18-20, 1090 Vienna, Austria; viktoria.koenig@meduniwien.ac.at; 2 Department of Plastic Surgery, MediCent Ostrava, Na Čtvrti 22, 70030 Ostrava-jih, Czech Republic; tomas.kempny@medicent.cz; 3 Department of Traumatology, University Hospital Brno and Faculty of Medicine, Masaryk University, 60177 Brno, Czech Republic; jakub.holoubek@med.muni.cz; 4 Department of Plastic Surgery, Hospital České Budějovice, 37001 Ceske Budejovice, Czech Republic; votruba.tomas@nemcb.cz; 5 Experimental Surgery, Third Faculty of Medicine, Charles University, 11000 Prague, Czech Republic; 6 Private Clinic—Spitalgasse 19, 1090 Vienna, Austria

**Keywords:** orthoplastic, lower limb reconstruction, free fibula, gunshot injuries, mandibula reconstruction, microsurgery, humanitarian mission, Tigray war

## Abstract

**Background:** Surgical care in conflict regions like Tigray, Ethiopia, faces severe challenges due to limited resources, infrastructural deficiencies, and high trauma burden. From 2019 to 2023, a multidisciplinary team conducted five humanitarian missions focusing on orthoplastic reconstruction of extremity and mandibular injuries from high-energy gunshot trauma. **Methods:** A retrospective analysis was performed on 98 patients who underwent free or pedicled flap reconstruction. Data included demographics, flap type, technique, complications, follow-up, and early clinical outcomes score as well as mobility scores. Flaps were harvested using loupes anastomosis performed using microscopes, depending on availability. **Results:** Among 98 patients (25.5% female, 74.5% male), 69 free flaps and 38 pedicled flaps were performed. Free fibula flaps (n = 54) included 33 mandibular and 21 extremity reconstructions. Additional flaps included ALT, gracilis, and LD flaps. Pedicled flaps included 18 fibula and 20 ALT/LD flaps. Mean age was 35.5 years; mean operative time was 429.5 min, with mandibular fibula transfers being longest. Microsurgical techniques were used in 34% of cases. Median follow-up was 10 months. Microsurgical complications occurred in 18.4%, mainly in fibula transfers (25.9%). Non-microsurgical issues included wound infections (n = 15), graft loss (n = 3), and bleeding (n = 5). Flap loss occurred in 16.3% overall. Early clinical outcome results were good (30.6%), acceptable (28.6%), and moderate (24.5%). **Conclusions:** Orthoplastic reconstruction using both free and pedicled flaps is feasible in low-resource, conflict settings. Despite infrastructural challenges, functional outcomes were achievable, supporting the value of adaptable microsurgical strategies in humanitarian surgery.

## 1. Introduction

Extremity reconstruction following high-energy trauma represents one of the most challenging and critical aspects of modern reconstructive surgery [1]. Complex limb injuries, particularly those involving extensive bone and soft tissue loss, often require advanced techniques such as free tissue transfer to restore form and function [2,3,4]. Free flaps, notably the free fibula flap, have become indispensable tools in orthoplastic surgery, offering robust vascularized bone suitable for the reconstruction of segmental defects [5,6,7,8]. Although originally pioneered in specialized centers of the developed world, microsurgical techniques have expanded significantly and are now utilized across multiple specialties including plastic surgery, orthopedics, otolaryngology, and neurosurgery [9,10,11,12].

Despite these advancements, a stark gap persists between developed and developing countries regarding the availability and outcomes of microsurgical interventions [6,12,13,14]. Particularly in sub-Saharan Africa, the access to and application of microsurgical free tissue transfer remains severely limited, often restricted to visiting surgical missions or a few pioneering local teams [6,12,13,14,15,16,17,18,19,20]. Factors such as inadequate infrastructure, scarcity of trained personnel, lack of essential equipment, and unpredictable perioperative support systems continue to hinder the routine practice of free tissue transfer in these regions [13,14,20]. Ironically, the burden of trauma and complex defects that require microsurgical reconstruction is often higher in low- and middle-income countries (LMICs) compared to high-income settings [11,14].

Tigray, the northernmost region in Ethiopia, exemplifies the compounded healthcare challenges faced in conflict-affected areas [19,20]. Historically underserved, the region’s healthcare system, already fragile, has been devastated by the ongoing civil conflict that erupted in November 2020 between the Ethiopian Federal Government and the Tigray Regional Government [19,20]. Widespread destruction of medical infrastructure, occupation and looting of health facilities, shortages of medical supplies, and mass displacement have led to a near-collapse of healthcare services [20]. Médecins Sans Frontières and other humanitarian organizations have reported that only a fraction of health facilities remain operational, with systematic attacks on medical services aggravating an already dire public health crisis [13,19].

Amid this humanitarian catastrophe, traumatic injuries, especially those caused by shotgun blasts and high-energy weapons, have become alarmingly prevalent [1]. These injuries often result in extensive soft tissue and bony defects that would ideally require complex reconstructive procedures, including vascularized bone grafting and free tissue transfer [18]. However, the realities on the ground—including frequent electricity outages, limited imaging, and constrained surgical support—present extraordinary barriers to delivering standard microsurgical care.

In response to this need, a multidisciplinary team of plastic and orthopedic surgeons conducted a series of humanitarian missions to Tigray, over a five-year period. The goal was to address severe extremity and maxillofacial injuries through orthoplastic strategies, with a strong focus on free fibula transfers for both limb salvage and mandibular reconstruction. Unlike typical short-term missions criticized for lack of sustainability, this initiative aimed to provide continuous support, training, and capacity-building alongside direct patient care [6].

The delivery of complex microsurgical procedures under these conditions demands not only technical proficiency but also logistical flexibility, resourcefulness, and deep cultural competence [6,12,13,14]. Recognizing the profound disparities in surgical care delivery globally, recent initiatives such as the Lancet Commission on Global Surgery and the Disease Control Priorities have emphasized the need to integrate essential surgical services into public health agendas [14]. As reconstructive surgery is an indispensable component of essential healthcare, the documentation of outcomes and challenges from missions like ours is critical to informing future strategies for surgical capacity-building in LMICs [13].

This study evaluates the outcomes of free and pedicled tissue transfers performed during humanitarian missions in Tigray, with a particular focus on extremity reconstruction following high-energy gunshot trauma. By analyzing clinical results, perioperative complications, and logistical challenges, we aim to contribute to the growing body of evidence on the feasibility, safety, and impact of orthoplastic microsurgery in severely resource-constrained and conflict-affected environments.

Ultimately, this work seeks to highlight both the potential and the limitations of advanced reconstructive surgery—particularly the use of free fibula flaps—in some of the world’s most challenging clinical environments.

## 2. Material & Methods

This study has been registered with ClinicalTrials.gov (ID: NCT06935916), approval date 5 May 2025. All medical records of a consecutive series of 98 patients who underwent free or pedicled tissue transfer after shotgun injuries between 2019 and 2023 were retrospectively reviewed. A subgroup analysis was performed for patients who received free tissue transfer for mandibular reconstruction.

Clinical data were collected to evaluate patient demographics (age, sex, mechanism of injury, side of injury), the type of tissue transfer performed, mean surgical time, and postoperative outcomes. Preoperative patient selection was conducted remotely in coordination with local healthcare providers and surgical staff on site. All patients who sustained gunshot injuries during the Tigray conflict and required reconstruction of either the mandible or an extremity using free or pedicled fibula flaps were considered for inclusion. Patient prioritization followed a pragmatic “first come, first served” principle, guided by clinical urgency and logistical feasibility.

Exclusion criteria included the unavailability of essential surgical materials (e.g., appropriate osteosynthesis plates or screws), lack of critical anesthetic equipment (such as pediatric endotracheal tubes), or limitations in operative capacity due to time constraints or mission resource exhaustion.

Microsurgical-related complications, including anastomosis revision, were assessed. Additionally, patient-related complications such as wound infection (superficial or deep), symptomatic pulmonary embolism, deep vein thrombosis (DVT), neurovascular injuries, and postoperative mortality were recorded.

All surgical procedures were conducted according to the local university hospital guidelines. Informed consent was obtained from all patients, with preoperative counseling carried out by local physicians who also provided translation support when necessary. Tissue flaps were raised using surgical magnifying loupes, irrigated with a heparinized saline solution, and a single intravenous dose of heparin was administered intraoperatively. Microsurgical anastomoses were performed using microsurgical sutures, and an operating microscope was utilized whenever possible; however, due to frequent electricity shortages, many procedures were completed using surgical loupes only.

Postoperatively, all patients were monitored in the intensive care unit (ICU) as clinically indicated. Flap monitoring was carried out regularly by trained nursing staff under direct medical supervision. Tracheostomies were performed if necessary prior to surgery. In cases where immediate reconstruction was pursued, the same surgical team typically conducted both the resection and the reconstruction.

## 3. Statistical Analysis

Statistical analysis was undertaken using R Version 3.1.1 SPSS (Armonk, New York, NY, USA).

Descriptive statistics (mean, median, range, and proportions) were used to summarize the patient cohort. Differences between means and proportions were analyzed using the chi-square test for categorical variables and the unpaired t-test for continuous variables. A *p*-value of ≤0.05 was considered statistically significant. Graphical data analysis and visualization were conducted with the assistance of ChatGPT 4 (OpenAI, 2024), using integrated data interpretation and plotting tools.

## 4. Results

**Figure 1:**
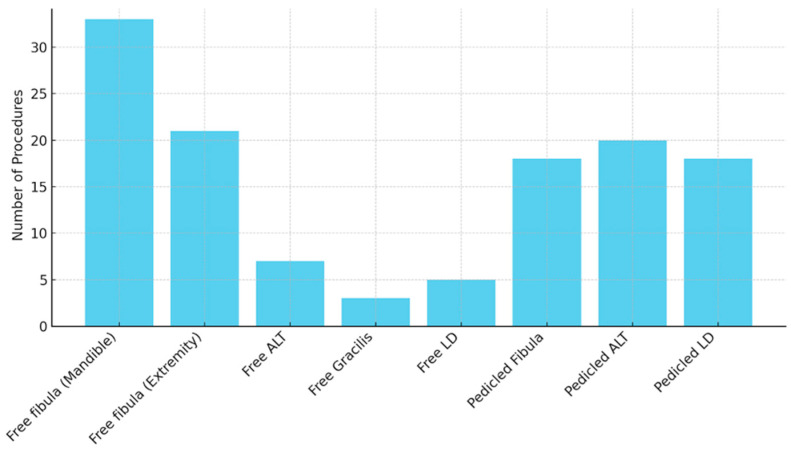
Flap type distribution.

A total of 98 patients underwent free tissue transfer following shotgun injuries, comprising 25 females (25.5%) and 73 males (74.5%). In total, 69 free flaps (70.4%) were performed. Of these, 54 (78.3% of free flaps) were free fibula transfers, further subdivided into mandibular reconstructions (n = 33, 61.1%) and extremity reconstructions (n = 21, 38.9%). Additional free flaps included anterolateral thigh (ALT) flaps (n = 7), M. gracilis muscle flaps (n = 3), and M. latissimus dorsi (LD) flaps (n = 5). Furthermore, 38 patients (38.8%) received pedicled flap reconstructions, consisting of pedicled fibula flaps (n = 18) and other pedicled ALT and LD flaps (n = 20) (Figure 1).

The mean patient age at the time of surgery was 35.5 years (range: 18–53 years). The mean operative time for all flap procedures was 429.5 min. Free fibula transfers for mandibular reconstruction had the longest mean operative time at 532.7 min (range: 480–600 min), compared to 480 min (range: 450–520 min) for extremity free fibula reconstructions. All surgeries were led by the visiting surgeon.

**Figure 2:**
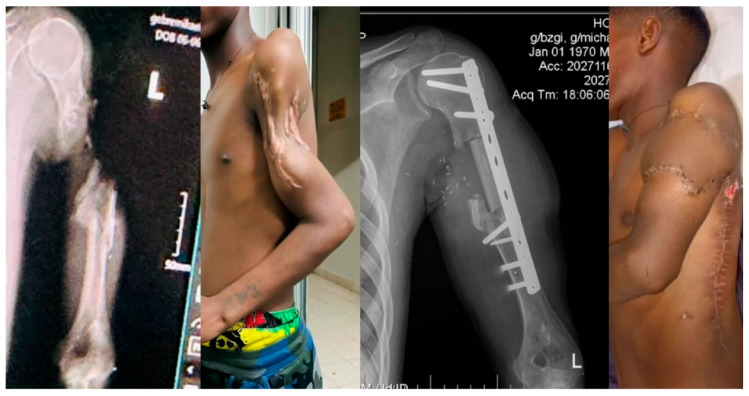
Outcome 3 months after upper limb reconstruction with free fibula transfer.

Standardized microsurgical techniques utilizing an operating microscope were employed in 34% of cases, while the remaining 66% were partially completed using surgical loupes due to frequent electricity shortages. Patients were followed up as outpatients for a median of 10 months (range: 3 weeks to 39 months). At the time of evaluation, 42% of patients remained under active follow-up care, while 25% of patients did not attend any postoperative outpatient appointments after discharge (Figure 2).

**Figure 3:**
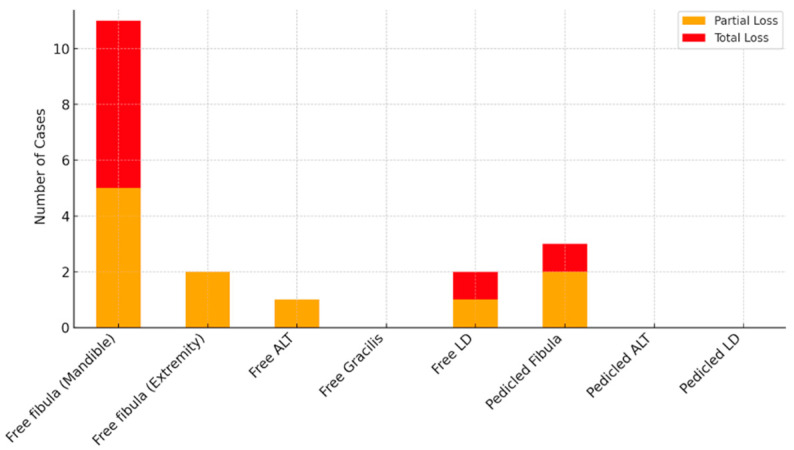
Flap loss rates by flap type.

Microsurgical complications related to anastomosis occurred in 18 cases (18.4%), with 14 (25.9%) involving free fibula transfers. Non-microsurgical complications were documented in 26.2% of patients, with wound infections occurring in 15 cases, graft loss in 3 cases, and postoperative bleeding in 5 cases. A total of 9 partial flap losses (9.2%) and 7 total flap losses (7.1%) were recorded. Notably, 6 out of 7 total free fibula graft losses (85.7%) occurred in mandibular reconstruction cases (Figure 3).

## 5. Functional Outcome

Among patients undergoing extremity reconstructions (n = 21), postoperative functional mobility was assessed using the Parker Mobility Score (PMS) and the Lower Extremity Functional Scale (LEFS) at three and six months follow-up. The mean PMS was 7.2 ± 1.8 at three months and improved to 8.4 ± 1.2 at six months (maximum score: 9), indicating a high level of regained mobility. The LEFS mean score was 56.7 ± 12.3 at three months and increased to 68.2 ± 9.8 at six months (maximum score: 80), suggesting moderate to good recovery of functional abilities in daily activities. Patients with successful free fibula transfers for extremity defects demonstrated significantly better PMS and LEFS scores compared to those reconstructed with pedicled flaps (*p* = 0.032).

**Figure 4:**
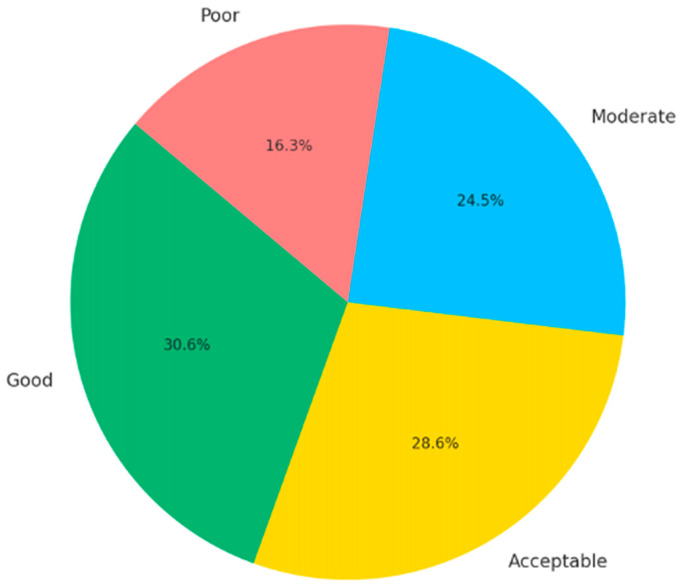
Early Clinical Outcome Score Distribution.

Evaluation of early clinical outcomes (Early Clinical Outcome Score) revealed the following distribution across all free and pedicled flap procedures: 30.6% of flaps achieved good results, 28.6% achieved acceptable outcomes, 24.5% demonstrated moderate results, and 16.3% experienced poor outcomes, including partial or total flap loss or major postoperative complications. When stratified by flap type, for pedicled fibula reconstructions, 33.3% of patients achieved good results, another 33.3% had acceptable outcomes, and 22.2% demonstrated moderate outcomes. For free fibula reconstructions, 18.5% of patients achieved good results, 29.6% had acceptable outcomes, and 27.8% showed moderate results (Figure 4).

## 6. Discussion

The free fibula flap (FFF) remains a cornerstone in mandibular and extremity reconstruction, offering vascularized bone with excellent mechanical properties—such as dense cortical structure, torsional resistance, and adaptability for osteotomies—alongside versatile soft tissue components [3,5,6,7,8,9,21,22,23,24]. In high-income countries, its success is often supported by virtual planning, stereolithographic modeling, and interdisciplinary collaboration [4,21,25]. In contrast, the humanitarian missions described in this study were conducted under extreme resource limitations—without intraoperative imaging, consistent electricity, or standard microsurgical equipment.

Despite these constraints, we achieved acceptable clinical outcomes. The fibula proved ideal for complex mandibular reconstructions, especially in cases requiring integrated soft tissue coverage and future implantability [2,3,25,26]. While non-vascularized bone grafts (NVBGs) are more accessible and technically less demanding, they are unreliable in large or irradiated defects and potentially unsuitable when simultaneous soft tissue reconstruction is necessary [7,27].

Su et al. confirmed the fibula flap’s role as the workhorse for mandibular reconstruction under ideal conditions [21]. Like Rodgers et al. stated, our missions’ success also depended on intraoperative adaptability, surgical experience, and structured teamwork [18]. Kadam et al. have emphasized the additional challenges of secondary reconstructions, such as fibrosis and distorted anatomy, which were also common in our cohort [10].

Compared to Rehman et al.’s reported 88.2% success rate for NVBGs in small defects (4–7 cm), our cohort involved more extensive defects and greater reconstructive complexity [27]. Banda et al. reported an 89% free flap survival rate in sub-Saharan Africa, with a 51% complication rate [11,12]. Our findings—showing a comparable complication profile and flap loss rate—mirror these challenges and underscore the importance of perioperative management in compromised settings. Walia et al. proposed the use of secondary free flaps after failure, yet this option remains largely theoretical in humanitarian missions due to logistical constraints [28].

Microsurgery in conflict-affected, resource-limited regions such as Tigray poses not only surgical but also ethical, logistical, and infrastructural challenges [6,11,12,13,14,15,16,17,18,19]. Major limitations included the absence of intraoperative imaging, frequent power outages, and the need to import essential materials such as microsurgical instruments and headlamps. Workaround protocols were developed to ensure sterilization and intraoperative flow.

Postoperative follow-up remains a persistent obstacle. Hendriks et al., in their review of 41 reconstructive missions, highlighted underreporting of complications due to follow-up loss [29]. Similarly, many of our patients could not be evaluated postoperatively. To address this, we implemented hybrid models involving local collaboration and remote consultation, with reassessment during follow-up missions whenever feasible.

The value of capacity-building was underscored by Citron et al., who documented improved flap survival (from 76% to 93%) at CoRSU Hospital in Uganda through structured training [6]. Our model similarly integrated clinical care, mentorship, and logistical assessment. A unique aspect of our cohort was the inclusion of patients with war-related injuries—an underrepresented group in global surgical literature.

Gebremariyam et al. emphasized the need for structured microsurgical programs in Ethiopia, aligning with our long-term vision of diagonal development missions [14]. While ongoing conflict and instability in Tigray currently limit such efforts, our repeated missions and the partnerships established with local teams lay a foundation for future progress.

A certain number of extremity reconstructions were performed, often after failed wound coverage at time of the accident. In these cases, the fibula flap proved equally valuable, particularly for segmental bone loss and chronic osteomyelitis. These results support its reliability beyond head and neck reconstruction, though prolonged operating times and challenging monitoring conditions remain limiting factors [21].

Our operative durations were consistent with findings by Citron et al., who reported a median of 508 min for fibula flaps due to complex mandibular contouring [6]. Our median time of 532.7 min reflects both the technical complexity and logistical constraints of microsurgery in humanitarian settings.

Beyond technical outcomes, this study contributes to the broader discourse on global reconstructive surgery. Sustainable care requires more than surgical expertise; it demands ethical frameworks, strategic planning, and consistent partnerships. De Berker et al. emphasized the dual potential of short-term missions to provide immediate care and foster long-term development, a model we aimed to replicate [13].

Based on five years of experience, we conclude that safe and effective surgical care in humanitarian missions requires careful attention to personnel selection, training, and structure. All participating surgeons were board-certified specialists with at least 6 to 7 years of post-residency experience in their respective fields, including plastic, maxillofacial, and orthopedic and trauma surgery. All plastic surgeons regularly performed free flap procedures in their home institutions. The local surgical team in Tigray included fully trained plastic surgeons as well as residents in training.

While humanitarian missions offer valuable learning opportunities, we strongly advocate that core team members possess independent microsurgical capability, clinical judgment, and the ability to adapt to resource-constrained environments. Only with such preparation can patient safety, surgical quality, and meaningful long-term outcomes be ensured in the complex setting of conflict-related humanitarian care.

## 7. Conclusions

Despite systemic instability and infrastructural limitations, complex microsurgical procedures—especially FFF for mandibular and extremity reconstruction—can be successfully performed in humanitarian missions when conducted by highly experienced teams. Patient safety, autonomy, and adaptability were central to our approach. Follow-up care remains a key challenge in these settings, often requiring creative hybrid strategies to ensure continuity. Short-term missions can bridge surgical gaps and contribute to local training, but sustainable outcomes require continued partnerships, diagonal development strategies, and long-term investment in global surgical infrastructure.

## 8. Limitations

This study has several limitations, primarily related to the challenging conditions under which the surgical missions were conducted. First, standardized operative conditions were not feasible due to frequent and unpredictable power outages, lack of intraoperative imaging, and limited availability of specialized microsurgical equipment. These factors prolonged operative times and necessitated constant intraoperative adaptability.

Second, follow-up was inconsistent and often incomplete. Due to the conflict setting and displacement of patients, many individuals could not return for postoperative evaluations. This significantly limits the ability to assess long-term outcomes, complication rates, and functional rehabilitation, particularly in cases requiring secondary interventions or implant-based dental reconstruction.

Third, patient selection and surgical planning were constrained by local infrastructure. Preoperative imaging was limited, remote planning depended on variable communication with local teams, and intraoperative decisions had to account for the availability of fixation materials, anesthesia resources, and operating room time.

The transferability of our results to other healthcare settings is inherently constrained. While the results provide insight into the feasibility of complex reconstructions in conflict-affected, resource-limited settings, they may not directly translate to other clinical environments.

## Data Availability

The data presented in this study are available on request from the corresponding author.
